# Nuclear ING2 expression is reduced in human cutaneous melanomas

**DOI:** 10.1038/sj.bjc.6603205

**Published:** 2006-06-06

**Authors:** F Lu, D L Dai, M Martinka, V Ho, G Li

**Affiliations:** 1 Department of Dermatology and Skin Science, Jack Bell Research Centre, Vancouver, BC, Canada V6H 3Z6; 2 Department of Pathology, Vancouver Coastal Health Research Institute, University of British Columbia, Vancouver, BC Canada V6H 3Z6

**Keywords:** ING2, tissue microarray, gene expression, melanoma

## Abstract

Cutaneous malignant melanoma is a severe and sometimes life-threatening cancer. The molecular mechanism of melanomagenesis is incompletely understood. Deregulation of apoptosis is probably one of the key factors contributing to the progression of melanoma. The inhibitor of growth (ING) family proteins are candidate tumour suppressors which play important roles in apoptosis. Downregulated expression of ING proteins have been reported in several tumour types, including the loss of nuclear expression of p33ING1b in melanoma. As ING2 exhibits 58.9% homology with p33ING1b, we hypothesized that the aberrant expression of ING2 may be involved in melanomagenesis. Here, we used tissue microarray technology and immunohistochemistry to examine ING2 expression in human nevi and melanoma biopsies. Our data showed that nuclear ING2 expression was significantly reduced in radial growth phase (RGP), vertical growth phase (VGP), and metastatic melanomas compared with dysplastic nevi (*P*<0.05). Our data also revealed that nuclear ING2 expression was not associated with patient's gender, age or tumour thickness, ulceration, American Joint Committee on Cancer (AJCC) stage, tumour subtype, location and 5-year survival (*P*>0.05). Taken together, our results suggest that nuclear ING2 expression is significantly reduced in human melanomas and that reduced ING2 may be an important molecular event in the initiation of melanoma development.

Human cutaneous malignant melanoma originates from the melanocytes of the skin or melanocytic lesions including common acquired nevus, dysplastic nevus, congenital nevus and cellular blue nevus. Once considered as an uncommon disease, the incidence of malignant melanoma has doubled within the last 10 years in the US (Balch *et al*, 2001). It is estimated that an American developing melanoma during his/her lifetime leaped from 1 in 1500 in 1960, to 1 in 68 in 2000, and is projected to increase to 1 in 50 by the year 2010 (Rigel, 2002; Dunlap *et al*, 2004). Primary melanoma usually progresses from the nonmetastatic radial growth phase to the vertical growth phase, in which the malignant cells invade the dermis and develop the ability to metastasize (Elder, 1999). The large majority of patients with early stages of melanoma can be cured by surgical removal while 50% of patients with distant metastasis die within the first 6 months after the diagnosis (Balch *et al*, 2001; Schoenlaub *et al*, 2001). Melanoma is highly resistant to conventional radio- or chemo-therapy. Dacarbazine (DTIC), the only FDA approved drug for treatment of melanoma, yields a response rate of only 16% (Atallah and Flaherty, 2005). Therefore, better understanding of the molecular mechanism of melanoma progression and chemoresistance is prompted for designing novel treatment regimes. P53, a central sensor linking DNA damage to apoptosis, plays an essential role in tumour suppression and chemosensitivity in many tumour types (Li *et al*, 1998; Raffo *et al*, 2000; Fridman and Lowe, 2003). However, mutational analysis reveals that the *p53* gene is altered in only approximately 11% of melanomas (Hussein, 2004). The low mutation rate of *p53* suggests that other tumour suppressor genes may play important roles in pathogenesis of melanoma.

Recent studies suggest that ING family proteins function as tumour suppressors. Five members of ING proteins have so far been identified and they all share a conserved PHD domain in the C-terminus (Campos *et al*, 2004). P33ING1b is the founding member which has been shown to induce cell cycle arrest, to enhance DNA repair and to promote apoptosis after DNA damage events (Garkavtsev *et al*, 1998; Cheung *et al*, 2001; Cheung and Li, 2002). ING2 was cloned through a homology search with p33ING1b and was found to be located to human chromosome 4 (Shimada *et al*, 1998). Besides, the common PHD zinc finger motif, ING2 also contains a nuclear localisation signal (NLS) domain and a unique leucine zipper domain that is thought to mediate hydrophobic protein–protein interaction (Feng *et al*, 2002). ING2 is mainly localised in the nucleus with 74% in the chromatin/nuclear matrix and 9% in the nucleoplasm in HT1080 fibrosarcoma cells (Gozani *et al*, 2003). Previous studies showed that ING2 cooperated with p53 to induce G_1_-phase cell-cycle arrest and enhance apoptosis in colorectal carcinoma RKO cells (Nagashima *et al*, 2001). Pedeux *et al* (2005) recently reported that ING2 regulates replicative senescence by cooperating with p300 to mediate p53 acetylation. In addition, ING2 was able to regulate nuclear response to DNA damage by interacting with phosphoinositides (PtdInsPs) through the PHD domain (Gozani *et al*, 2003).

We have recently demonstrated that ING2 plays an essential role in cellular stress response to UV irradiation either by enhancing nucleotide excision repair or promote UV-induced apoptosis in melanoma cell lines (Chin *et al*, 2005; Wang *et al*, 2006). To further investigate the role of ING2 in melanomagenesis, we used tissue microarray (TMA) and immunohistochemistry to evaluate the expression of ING2 in different stages of human melanocytic lesions. Our results for the first time showed that nuclear ING2 expression was reduced in melanoma biopsies compared to dysplastic nevi.

## MATERIALS AND METHODS

### TMA construction

Formalin-fixed, paraffin-embedded tissue blocks containing normal nevi, dysplastic nevi, primary melanomas and metastatic melanomas were used in this study. The diagnosis of melanocytic lesions followed criteria from other groups (Keller and Maize, 1996; Metcalf, 1996). Sample collection was approved by the University of British Columbia and was performed in accordance with the Declaration of Helsinki Guidelines. All the tissue samples were from 1990 to 1997 archives of the Department of Pathology, Vancouver General Hospital. For each case, the most representative lesion area was selected and marked on haematoxylin–eosin-stained slides. Taking into account the limitations of the representative areas of the tumour, duplicate 0.6-mm-diameter tissue cores were taken from each biopsy and the TMAs were assembled using a tissue array instrument (Beecher Instruments, Silver Spring, MD, USA). Multiple 4-*μ*m sections were cut with a Leica microtome and then transferred to adhesive-coated slides using routine histology procedures. One section from each TMA was routinely stained with haematoxylin and eosin. The remaining sections were stored at room temperature for immunohistochemistry staining.

### Immunohistochemistry of TMA

The TMA slides were deparaffined by heating at 55°C for 30 min following by three washes with xylene, 5 min each. Tissues were then rehydrated in a series of 5-min washes in 100, 90 and 70% ethanol and rinsed with phosphate-buffered saline (PBS). Antigen retrieval was performed by microwaving the samples for 4 min at full power (800 W) in 250 ml of 10 mM sodium citrate (pH 6.0). Endogenous peroxidase activity was blocked with 0.3% hydrogen peroxide for 20 min and nonspecific binding was blocked by universal blocking serum (DAKO Diagnostics, Mississauga, Ontario, Canada) for 30 min. The primary polyclonal rabbit anti-ING2 antibody Ping2, a kind gift of Dr C Harris (NIH, Bethesda, MD, USA), was diluted 1 : 250 and incubated at 4°C overnight. After three washes, 2 min each with PBS, the sections were incubated with a biotinylated goat anti-rabbit secondary antibody for 30 min (Santa Cruz Biotechnology, Santa Cruz, CA, USA). After three washes with PBS for 2 min each, horseradish peroxidase-streptavidin (Santa Cruz Biotechnology) was added to the section for 30 min, followed by another three washes, 2 min each with PBS. The samples were developed with 3,3-diaminobenzidine substrate (Vector Laboratories, Burlington, Ontario, Canada) for 7 min and counterstained with haemotoxylin. Then the slides were dehydrated following a standard procedure and sealed with coverslips. Negative controls were performed by omitting ING2 antibody during the primary antibody incubation.

### Evaluation of immunostaining

ING2 staining intensity was evaluated blinded by three independent observers (including one dermatopathologist) simultaneously, and a consensus score was reached for each core. In all, 11 normal nevi and 57 dysplastic nevi were evaluated for ING2 staining, and informative tumour staining and complete clinical pathological information were obtained in 79 primary melanoma cases and 43 metastatic melanoma cases. The staining intensity was scored as negative (0), weak (1), moderate (2) and strong (3). For ING2 staining intensity, 87% of the biopsies have uniform staining between different cores. In the 13% cases with a discrepancy between duplicated cores, the average score was obtained. In addition, the percentage of cells showing staining in the nucleus was assessed by counting a minimal 400 cells per tissue core and the average percentage of duplicate biopsy cores was calculated. The multiplication of the average intensity score and the average percentage was used as the final staining score for statistical analysis.

### Statistical analysis

Nuclear ING2 expression among different melanocytic lesions as well as its correlation with clinicopathological parameters of the melanoma patients, including age, gender, tumour thickness, location, histological subtype, tumour ulceration status and AJCC staging was evaluated by Mann–Whitney test or Kruskal–Wallis test. Survival curves were plotted according to Kaplan–Meier method and the comparison of survival curves was performed with the log-rank test. The Mann–Whitney and Kruskal–Wallis tests were performed by using GraphPad Prism and the survival analysis was performed with SPSS 11.5 (SPSS, Chicago, IL, USA). A *P*-value <0.05 was considered significant.

## RESULTS

### Clinicopathological findings

The clinicopathological features of the melanomas for this study are summarised in Table 1. In primary melanomas, 79 cases (50 male subjects and 29 female subjects) were available for the evaluation of ING2 staining. The median age of the patients was 58 y ranging from age 25 to 92 years. There were 12 cases of RGP and 67 cases of VGP. For the thickness of these primary melanomas, 23 were ⩽1.0 mm, 27 were 1.01–2.0 mm, 15 were 2.01–4.0 mm and 14 were >4 mm. For the tumour subtype, superficial spreading melanoma accounted for 36 cases, lentigo maligna melanoma 13 cases, acrolentigous melanoma two cases, nodular melanoma 13 cases and the remaining 15 cases were unspecified. The majority of the melanomas located in sun-protected sites (65 cases, trunk, arm, leg and feet) while 14 located in sun-exposed sites (head and neck). Tumour ulceration was found in 16 patients. Forty-three out of 50 metastatic melanoma cases were available for ING2 staining. There are 29 male and 14 female, with age ranging from 27 to 89 (median 59 years). We also used AJCC criteria to evaluate ING2 expression among 122 melanomas (79 primary melanomas and 43 metastatic melanomas), 41 tumours were at stage I, 34 at stage II, 24 at stage III and 23 at stage IV.

Clinically, dysplastic nevi can be identified as mild, moderate or severe. While mildly and moderately dysplastic nevi can be closely observed, severely dysplastic nevi should certainly be surgically removed. In this study, information on 53 of 57 dysplastic nevi are available for subcategorization: 22 mild, 20 moderate and 11 severe.

### Reduced ING2 nuclear expression in human melanomas

**Figure 1:**
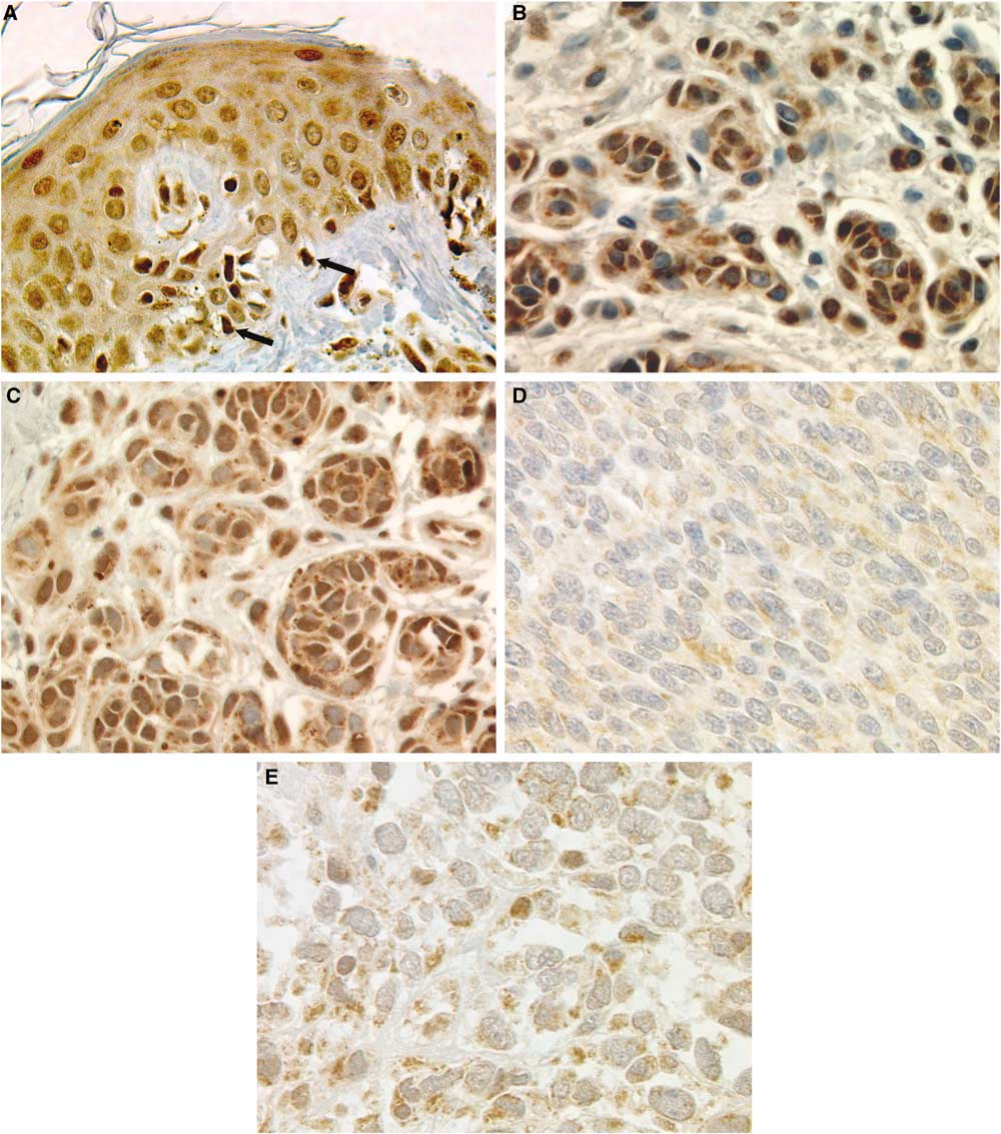
Representative images of ING2 immunohistochemical staining in human melanocytic lesions. Strong ING2 expression in adjacent normal epidermis (**A**), normal nevi (**B**), dysplastic nevi (**C**), and weak ING2 staining in primary melanoma (**D**) and metastatic melanoma (**E**). Arrows indicate strong staining in melanocyte. Magnification, × 400.

**Figure 2:**
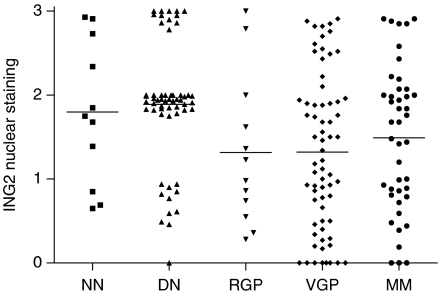
ING2 nuclear expression at different stages of melanocytic lesions. There are less ING2 nuclear expression in RGP, VGP and metastatic melanomas compared with dysplastic nevi (*P*=0.029, 0.0001 and 0.0286, respectively, Mann–Whitney test).

We examined ING2 nuclear expression in normal nevi, dysplastic nevi, primary melanomas and metastatic melanomas by immunohistochemistry (Figure 1). There is no significant difference in ING2 nuclear expression between normal nevi and dysplastic nevi (*P*>0.05, Mann–Whitney test). No difference in ING2 expression was found among mildly, moderately or severely dysplastic nevi (*P*>0.05, Kruskal–Wallis test). Reduced ING2 expression was observed in RGP and VGP primary melanomas as well as metastatic melanomas compared with dysplastic nevi (*P*=0.029, 0.0001 and 0.0286, respectively, Mann–Whitney test). There are no significant differences in ING2 nuclear expression among RGP, VGP and metastatic melanomas (*P*>0.05, Kruskal–Wallis test) (Figure 2).

### Correlation between ING2 nuclear expression and clinicopathological parameters or 5-year patient survival

**Figure 3:**
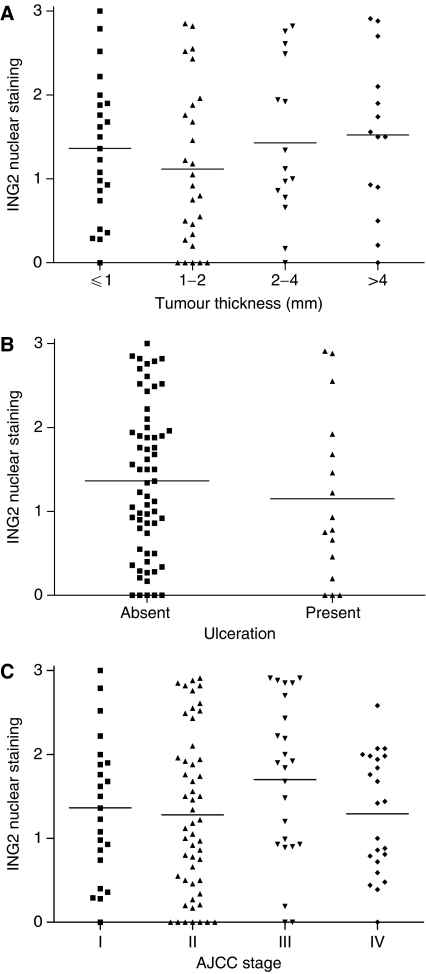
No correlation was found between ING2 nuclear expression and tumour thickness (*P*>0.05, Kruskal–Wallis test) (**A**) and tumour ulceration (*P*>0.05, Mann–Whitney test) (**B**) of primary melanomas, or AJCC stages for all 122 melanoma cases (*P*>0.05, Kruskal–Wallis test) (**C**).

**Figure 4:**
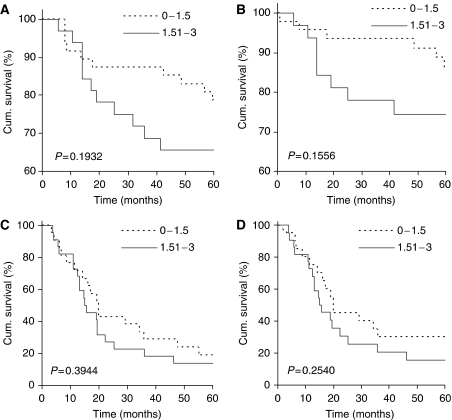
ING2 nuclear expression and 5-year patient survival. ING2 nuclear expression is not correlated with 5-year overall (**A**, **C**) and disease-specific survival (**B**, **D**) in primary melanoma patients (**A**, **B**) or metastatic melanoma patients (**C**, **D**).

Tumour thickness, ulceration and AJCC stages are well-known indicators for melanoma prognosis. However, no correlation was found between ING2 nuclear staining and these parameters (*P*>0.05 for all) (Figure 3). In addition, no association was found between ING2 nuclear expressions and other clinicopathological parameters including age, gender, subtype and location of tumours (data not shown). To investigate whether ING2 expression was correlated with patient survival, Kaplan–Meier survival curves were plotted to see if there was a relationship between ING2 nuclear expression and 5-year patient survival in primary melanomas or metastatic melanomas. We defined the staining as strong if the score is between 1.51 and 3.0 or weak if the score is ⩽1.5. Our results showed that ING2 nuclear expression did not significantly correlate with both 5-year overall and disease-specific patient survival in primary and metastatic melanomas (*P*>0.05, log-rank test) (Figure 4).

## DISCUSSION

The main purpose of this study is to investigate if the novel tumour suppressor ING2 is aberrantly expressed in human cutaneous melanomas. Using the tissue microarray technology and immunohistochemistry, we for the first time demonstrated that ING2 nuclear expression is reduced in human melanomas compared to dysplastic nevi (Figure 2). Although a number of studies indicated that ING2 possesses tumour suppressive functions, such as inducing growth arrest, senescence, apoptosis and enhancing DNA repair (Nagashima *et al*, 2001; Gozani *et al*, 2003; Chin *et al*, 2005; Pedeux *et al*, 2005; Wang *et al*, 2006), there is limited information on ING2 expression level in human cancers. There is only one recent report by Okano *et al* (2006) showing that ING2 expression is reduced in six of seven lung cancer cell lines. However, these authors did not detect any *ING2* mutation in 31 human lung cancer cell lines and 30 lung cancer biopsies. Although the reason for reduced ING2 expression in human melanomas is unclear, we cannot rule out mutation of the *ING2* gene in melanoma because different tumour type may have different mechanisms for gene inactivation. For example, reduced ING1 expression was found in 73% of non-small cell lung cancer biopsies (Kameyama *et al*, 2003) and 44% of breast cancer primaries (Toyama *et al*, 1999), while no missense mutation were found in these lung cancer biopsies and only one missense mutation was found in 377 breast cancer carcinomas (0.27%). On the other hand, reduced nuclear ING1 expression was associated with a much higher mutation rate (20%) of the *ING1* gene in human primary melanomas (Campos *et al*, 2004). In addition, loss of heterozygosity of the region 4q32 in the long arm of chromosome 4, which includes ING2 and SAP30, was found in 20% of basal cell carcinomas (Sironi *et al*, 2004), suggesting that genetic alteration of the ING2 gene does occur in human tumours. Future studies on ING2 expression and gene mutation in different cancer types will provide further evidence on the important role of this tumour suppressor in the pathogenesis of human cancers.

Similar levels of ING2 nuclear expression in melanomas regardless of the growth phases (RGP vs VGP) (Figure 2), tumor thickness, ulceration or AJCC staging (Figure 3) suggest that reduced ING2 expression may be involved in the initiation, rather than progression of melanoma. We have recently shown that ING2 plays an essential role for maintaining genomic stability upon UV irradiation. ING2 acts as a DNA damage sensor for nucleotide excision repair, as physiological level of ING2 is required for rapid induction of histone H4 acetylation, chromatin relaxation and the recruitment of repair recognition factor XPA to the photolesion sites (Wang *et al*, 2006). ING2 can also enhance the removal of DNA damage by triggering the apoptosis process when the DNA damage is too severe. We have found that overexpression of ING2 significantly enhanced UV-induced apoptosis by downregulating the expression of Bcl-2, promoting the translocation of the Bax protein to the mitochondria, resulting in the mitochondrial membrane potential change, release of cytochrome *c* and activation of caspases-9 and -3 (Chin *et al*, 2005). As UV radiation is the main environment factor for melanoma formation, reduced ING2 expression would impair the removal of UV-damaged DNA, leading to gene mutation and malignant cell transformation.

Our data that no significant correlation between ING2 expression and melanoma thickness or AJCC staging supports a multiple genetic change model during melanoma pathogenesis. Many different molecules in the apoptosis and survival pathways have been shown to be involved in melanoma initiation and progression. It appears that defects in the apoptosis pathway mostly attribute to the initiation step in melanoma pathogenesis, while activated survival pathway often correlates with tumour progression. For example, the expression of proapoptotic factor Apaf-1, a p53 downstream effector, which links the release of cytochrome *c* to the activation of caspase-9, is reduced in human cutaneous melanomas compared with normal nevi (Dai *et al*, 2004). The expression of another p53 downstream effector PUMA is significantly reduced in melanoma compared to dysplastic nevi (Karst *et al*, 2005). Furthermore, the expression of proapototic protein XAF1, which blocks the XIAP-mediated inhibition of caspase-3 (Liston *et al*, 2001), is also reduced in human melanomas (Ng *et al*, 2004). However, the expression of Apaf-1, PUMA and XAF1 expression did not correlate with tumor thickness. On the other hand, increased expression of integrin-linked kinase and phospho-Akt in the PI3 kinase survival pathway has been observed in melanoma and correlated with tumour thickness and 5-year patient survival (Dai *et al*, 2003, 2005). Furthermore, reduced expression of PTEN, the negative regulator of the PI3 kinase, was also found to be reduced in melanoma and significantly associated with tumour thickness (Goel *et al*, 2006). However, some factors are involved in more than one step in melanoma pathogenesis. For instance, although PUMA expression does not correlate with tumour thickness in primary melanoma, it is further reduced in metastatic melanoma and weaker PUMA expression is correlated with a poorer 5-year patient survival (Karst *et al*, 2005). Based on the complexity of the apoptotic and survival pathways which govern the fate of a cell, additional studies on the timing of the gene inactivation/overexpression in these pathways from the same set of tumour biopsies and the interdependence among these events will provide a more complete picture of the molecular changes during melanoma pathogenesis.

Our data that reduced ING2 expression does not significantly correlate with 5-year patient survival (Figure 4) is consistent with the findings that ING2 reduction is an early event in the RGP and does not correlate with tumour progression (Figures 2 and 3). On the other hand, although it is not statistically significant, we observed that patients with weaker ING2 expression have a trend towards better prognosis for both overall and disease-specific 5-year patient survival in primary melanomas (Figure 4). Interestingly, decreased expression of p33ING1b was also associated with better prognosis in childhood acute lymphoblastic leukemia patients as well as in invasive bladder cancer (Nouman *et al*, 2002; Sanchez-Carbayo *et al*, 2003). Although the mechanism underlying these phenomena is unclear, we propose that the enhancement of DNA repair by ING2 (Wang *et al*, 2006) may account for a better trend of 5-year patient survival in weaker ING2 expression group because enhanced DNA repair have been shown to reduce the cytotoxicity of anticancer drugs (Siddik, 2003). Future studies on the correlation between ING2 expression and patient survival in a large set of melanoma biopsies from patients with and without chemotherapy will provide a clearer answer.

In summary, we found that ING2 nuclear expression is reduced in human cutaneous melanomas compared to normal or dysplastic nevi. These data, together with our previous findings that ING2 plays essential roles in cellular stress responses (Chin *et al*, 2005; Wang *et al*, 2006), suggest the importance of this tumour suppressor in the pathogenesis of melanoma.

## Figures and Tables

**Table 1:** Clinicopathological parameters of 122 cases of melanomas

	**No. of Patients**	**%**
*Primary melanoma*		
*Age*		
⩽58	38	48
>58	41	52
		
*Gender*		
Male	50	63
Female	29	37
		
*Tumour thickness (mm)*		
⩽1	23	29
1.01–2	27	34
2.01–4	15	20
>4	14	17
		
*Ulceration*		
Absent	63	80
Present	16	20
		
*Tumour subtype*		
Superficial spreading melanoma	36	46
Lentigo maligna melanoma	13	16
Acrolentigous melanoma	2	3
Nodular melanoma	13	16
Unspecified	15	19
		
*Tumour growth phase*		
Radial growth phase	12	15
Vertical growth phase	67	85
		
*Site* a		
Sun-protected	65	82
Sun-exposed	14	18
		
*Metastatic melanoma*		
*Age*		
⩽59	22	51
>59	21	49
		
*Gender*		
Male	29	67
Female	14	33
		
*AJCC Stage*		
I	41	34
II	34	28
III	24	20
IV	23	18

a Sun-protected sites: trunk, arm, leg and feet. Sun-exposed sites: head and neck.
